# Reappraisal of XRCC1 Arg194Trp polymorphism and glioma risk: a cumulative meta-analysis

**DOI:** 10.18632/oncotarget.15376

**Published:** 2017-02-16

**Authors:** Jun-Ti Lu, Ai-Ping Deng, Juan Song, Li Zhang, Jie Luo

**Affiliations:** ^1^ Department of Neurosurgery, Taihe Hospital, Hubei University of Medicine, Shiyan 442000, China; ^2^ Department of Clinical Laboratory, Taihe Hospital, Hubei University of Medicine, Shiyan 442000, China

**Keywords:** XRCC1, X-ray repair cross-complementing group 1, polymorphism, glioma, meta-analysis

## Abstract

The association between XRCC1 Arg194Trp polymorphism and glioma risk were inconsistent from published meta-analyses and epidemiological studies. Hence, we performed this updated and cumulative meta-analysis to reappraisal this relationship. PubMed, Embase, CBM (Chinese Biomedical Database), and CNKI (China National Knowledge Internet) databases were comprehensively searched up to August 13, 2016 (updated on December 22, 2016). After study selection and data extraction from eligible studies, the association was evaluated by odds ratios (ORs) and its 95% confidence intervals (95%CIs) using Comprehensive Meta-Analysis software. Finally 16 case-control studies involving 7011 patients and 9519 healthy controls were yielded. The results indicated that XRCC1 Arg194Trp polymorphism was significantly correlated with the increased risk of glioma [Trp vs. Arg: OR = 1.18(1.05-1.34); TrpTrp vs. ArgArg: OR = 1.66(1.31-2.12); ArgTrp vs. ArgArg: OR = 1.34(1.02-1.77); TrpTrp vs. ArgArg+ArgTrp: OR = 1.47(1.26-1.72); TrpTrp+ArgTrp vs. ArgArg: OR = 1.17(1.01-1.35)]. Cumulative analysis showed the results changed from non-significant to significant when new studies accumulated, and sensitivity analysis indicated the results were stable. Subgroup analysis showed the significant association existed in Asians but not in Caucasians. Current evidence indicated that XRCC1 Arg194Trp polymorphism was associated with increased risk for glioma, especially in Asians; however, relevant studies involving other ethnic groups are required to validate our findings in further.

## INTRODUCTION

Glioma is the most common and the worst prognosis on primary central nervous system (CNS) tumors, making up approximately 30 % of all brain and CNS tumors and 80 % of all malignant brain tumors [1, 2]. However, the etiology of glioma is largely unknown. The radiation exposure and certain genetic syndromes are well-defined risk factors for malignant glioma [3]. The base excision repair (BER), nucleotide excision repair (NER), mismatch repair (MMR), and double strand break repair (DSBR) are the four major DNA repair pathways [4]. X-ray repair cross-complementing group 1 (XRCC1) gene, which is located on chromosome 19q 13.2–13.3 with a length of 33 kilobases, is one of the DNA repair genes encoding a scaffolding protein that participates in BER pathway [5, 6]. There are more than 300 validated single nucleotide polymorphisms (SNPs) in the XRCC1 gene in the dbSNP database; thereinto, Arg399Gln (rs25487), Arg280His (rs25489), and Arg194Trp (rs1799782) are the three extensively studied polymorphisms.

From 2012 to 2016, there are 14 meta-analyses [7–20] published to estimate the association between XRCC1 Arg194Trp polymorphism and glioma risk, but the results are contradictory (Supplementary Table 1). Hence, it is still unclear whether XRCC1 Arg194Trp polymorphism is associated with risk of glioma. In 2014, Adel Fahmideh et al [21] conducted a systematic review and meta-analysis to investigate the association between DNA repair gene polymorphisms (ERCC1 rs3212986, ERCC2/XPD rs13181, MGMT rs12917, PARP1 rs1136410, and XRCC1 rs25487) and risk of glioma. Obviously, XRCC1 rs1799782 polymorphism is not included. Moreover, there are many relevant studies which are published after the previous 14 meta-analyses and also yield inconsistent results. Therefore, we performed this updated and cumulative meta-analysis [22, 23] to explore the more precise association between Arg194Trp polymorphism and glioma risk. Subgroup analyses were also performed according to Caucasian and Asian populations to investigate ethnicity-specific effects; the subgroup analyses based on the source of controls and the HWE for controls were conducted as well.

## RESULTS

### Study selection and characteristics

**Figure 1:**
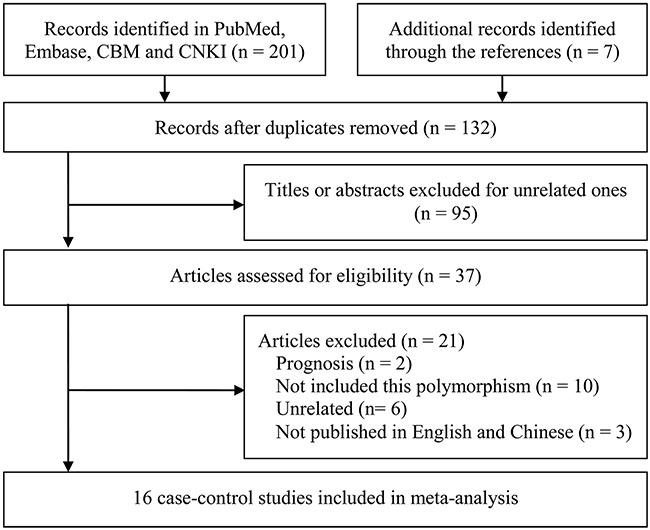
Flow chart from identification of eligible studies to final inclusion

The primary search yielded 208 potentially related publications and finally 16 case-control studies involving 7011 patients and 9519 healthy controls were included [24–39]. Figure 1 shows the study selection process. Of these studies, five dealt with probands of Caucasian origin [24–27, 30] and eleven referred to Asian origin [28, 29, 31–39]; five studies were out of Hardy Weinberg Equilibrium (HWE) [28, 30, 31, 34, 37]. Table 1 lists the main characteristics of identified studies.

**Table 1:** Characteristics of included studies in the meta-analysis

References	Country (Ethnicity)	Case				Source of control	Control				Genotyping method	HWE
		Total	CC	CT	TT		Total	CC	CT	TT
Liu 2007	China (Asian)	756	371	308	77	Mixed	754	375	305	74	TaqMan	Yes
Kiuru 2008	Europeancountries(Caucasian)	700	626	71	3	PB	1556	1377	177	2	PCR-RFLP	Yes
Liu 2009	USA (Caucasian)	210	180	29	1	PB	365	310	52	3	MassARRAY	Yes
Mckean-Cowdin 2009	USA (Caucasian)	1022	842	177	3	Mixed	2022	1664	352	6	TaqMan	Yes
Rajaraman 2010	USA (Caucasian)	342	304	38	0	HB	468	394	73	1	TaqMan	Yes
Hu 2011	China (Asian)	127	71	38	18	HB	249	163	64	22	PCR-CTPP	No
Zhou 2011	China (Asian)	271	145	112	14	HB	289	159	117	13	TaqMan	Yes
Custodio 2011	Brazil (Caucasian)	80	15	31	34	PB	100	67	4	29	PCR-RFLP	No
Wang 2012	China (Asian)	624	376	218	30	HB	580	355	205	20	PCR-RFLP	Yes
Liu 2012	China (Asian)	444	294	105	45	HB	442	334	89	19	MassARRAY	No
Luo 2013	China (Asian)	297	204	63	30	HB	415	297	96	22	MassARRAY	Yes
Pan 2013	China (Asian)	443	301	116	27	HB	443	327	101	15	MassARRAY	No
Xu 2014	China (Asian)	886	525	301	60	HB	886	540	311	35	PCR-RFLP	Yes
Gao 2014	China (Asian)	326	235	73	18	HB	376	279	84	13	MassARRAY	No
Li 2014	China (Asian)	368	183	171	16	HB	346	175	151	20	PCR-RFLP	Yes
Fan 2016	China (Asian)	115	31	58	26	HB	228	82	109	37	PCR-RFLP	Yes

PB, population-based; HB, hospital-based; Mixed, population and hospital based; HWE, Hardy Weinberg Equilibrium

### Meta-analysis

**Figure 2:**
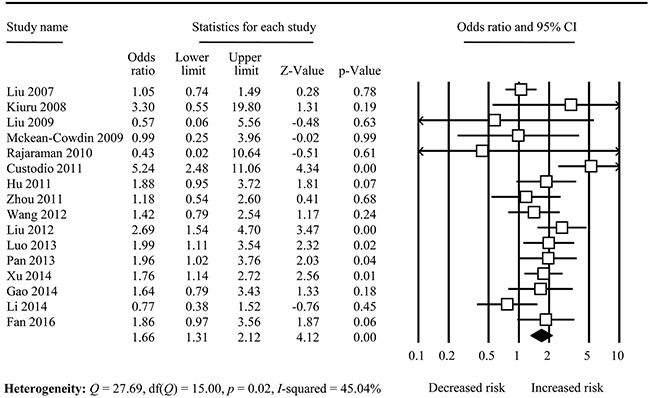
Forest plot for overall analysis in TrpTrp vs. ArgArg comparison

Table 2 demonstrates the results of overall and subgroup analyses. Overall, XRCC1 Arg194Trp polymorphism was significantly associated with increased risk of glioma under all five genetic models: the allele comparison, homozygote comparison, heterozygote comparison, recessive model, and dominant model [for Trp vs. Arg: odds ratio (OR) and its 95% confidence interval (95%CI) = 1.18(1.05-1.34), *I*^2^= 73.96%; for TrpTrp vs. ArgArg: OR = 1.66(1.31-2.12), *I*^2^ = 45.84%, Figure 2; for ArgTrp vs. ArgArg: OR = 1.34(1.02-1.77), *I*^2^ = 91.01%; for TrpTrp vs. ArgArg+ArgTrp: OR = 1.47(1.26-1.72), *I*^2^ = 13.64%; TrpTrp+ArgTrp vs. ArgArg: OR = 1.17(1.01-1.35), *I*^2^ = 72.60%, respectively].

**Table 2:** Results of overall and subgroups analyses of pooled ORs and 95% CIs

	No.	Trp vs. Arg			TrpTrp vs. ArgArg			ArgTrp vs. ArgArg			TrpTrp+ArgTrp vs. ArgArg			TrpTrp vs. ArgArg+ArgTrp		
		OR (95%CI)	*p* for OR	*I*^2^(%)	OR (95%CI)	*p* for OR	*I*^2^(%)	OR (95%CI)	*p* for OR	*I*^2^(%)	OR (95%CI)	*p* for OR	*I*^2^(%)	OR (95%CI)	*p* for OR	*I*^2^(%)
**Overall**	16	1.18 (1.05-1.34)	0.01	73.96	1.66 (1.31-2.12)	0.04	45.84	1.34 (1.02-1.77)	<0.01	91.01	1.17 (1.01-1.35)	0.04	72.60	1.47 (1.26-1.72)	<0.01	13.64
**Ethnicity**																
Asian	11	1.20 (1.08-1.32)	<0.01	47.86	1.52 (1.28-1.80)	0.13	34.10	1.36 (0.97-1.91)	0.07	91.64	1.14 (1.05-1.24)	<0.01	11.42	1.46 (1.24-1.72)	<0.01	31.72
Caucasian	5	1.15 (0.74-1.77)	0.54	89.03	1.93 (0.69-5.36)	0.21	50.25	1.31 (0.77-2.23)	0.32	89.6	1.27 (0.76-2.12)	0.37	90.3	1.59 (0.95-2.67)	0.08	0
**Source of controls**																
Hospital	11	1.19 (1.06-1.34)	<0.01	57.30	1.69 (1.40-2.05)	0.41	2.92	1.32 (0.91-1.91)	0.14	91.96	1.15 (1.02-1.30)	<0.01	39.80	1.62 (1.34-1.95)	<0.01	1.42
Population	3	1.45 (0.63-3.35)	0.38	92.94	4.08 (2.11-7.90)	<0.01	40.26	2.58 (0.70-9.44)	0.15	94.3	1.88 (0.60-5.86)	0.28	94.4	1.79 (1.02-3.16)	0.04	0
Mixed	2	1.01 (0.90-1.14)	0.84	0	1.05 (0.75-1.47)	0.79	0	1.01 (0.87-1.16)	0.93	0	1.01 (0.88-1.16)	0.89	0	1.04 (0.75-1.44)	0.82	0
**HWE**																
Yes	11	1.05 (0.98-1.13)	0.13	16.78	1.34 (1.11-1.63)	0.36	8.87	1.20 (0.86-1.68)	0.29	92.48	1.02 (0.94-1.11)	0.58	0	1.31 (1.09-1.58)	<0.01	10.13
No	5	1.65 (1.23-2.22)	<0.01	79.36	2.41 (1.79-3.65)	0.18	35.78	1.79 (1.06-3.05)	0.03	87.21	1.81 (1.16-2.83)	0.01	85.86	1.93 (1.45-2.56)	<0.01	0

Egger’s test: 0.22; 0.83; 0.12; 0.06; 0.97

**Figure 3:**
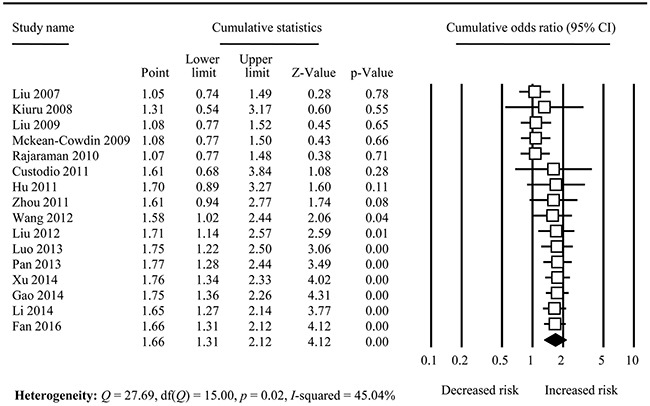
Forest plot for cumulative analysis in TrpTrp vs. ArgArg comparison

**Figure 4:**
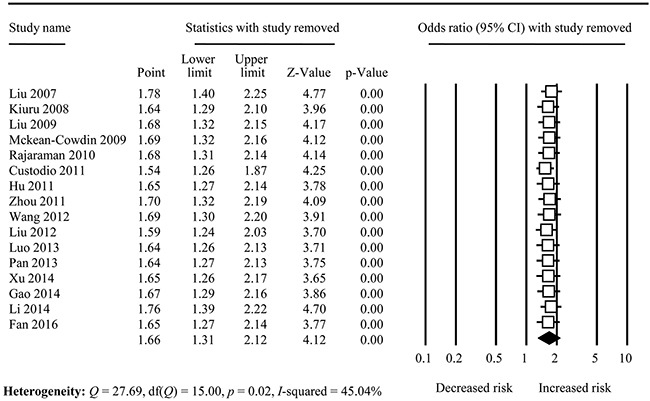
Forest analysis for sensitivity analysis in TrpTrp vs. ArgArg comparison

The cumulative meta-analysis accumulated the studies according to the publication year which displayed that the association change from non-significant to significant with new studies accumulated, and the CIs became increasing narrower (Figure 3 and Supplementary Figure 1 to Figure 4). Sensitivity analysis indicated that the overall analysis was not influenced by any single study (Figure 4).

In the subgroup analysis for ethnicity, no significant association was found in Caucasians under all five genetic models, but significantly increased risk was observed in Asians under for contrasts (Trp vs. Arg, TrpTrp vs. ArgArg, TrpTrp+ArgTrp vs. ArgArg, and TrpTrp vs. ArgArg+ArgTrp). After stratified analysis by source of controls, significant results were found both in hospital -based (Trp vs. Arg, TrpTrp vs. ArgArg, TrpTrp+ArgTrp vs. ArgArg, and TrpTrp vs. ArgArg+ArgTrp) and significant results for population-based controls (TrpTrp vs. ArgArg). Significant association existed in the studies conforming to HWE under two genetic models (TrpTrp vs. ArgArg and TrpTrp vs. ArgArg+ArgTrp) and in the studies deviating from HWE under all five genetic models.

### Publication bias

**Figure 5:**
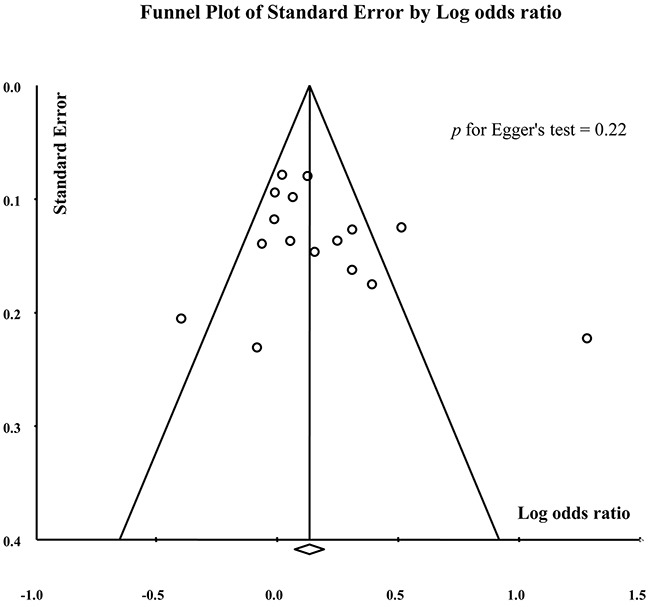
Funnel plot for the assessment of publication bias in Trp vs. Arg comparison

As shown in Figure 5, no obvious publication bias was found. The Egger’s test also showed no evidence of publication bias (Trp vs. Arg: p = 0.22; TrpTrp vs. ArgArg: p= 0.83; ArgTrp vs. ArgArg: p = 0.12; TrpTrp+ArgTrp vs. ArgArg: p = 0.06; TrpTrp vs. ArgArg+ArgTrp: p = 0.97).

## DISCUSSION

The first study on the association between XRCC1 Arg194Trp polymorphism and glioma risk was performed by Liu et al in 2007 [36], involving 756 cases and 754 controls from Chinese, and the results indicated no significant association between XRCC1 Arg194Trp polymorphisms and glioma risk. In 2012, Zhang et al conducted a meta-analysis to explore the role of XRCC1 Arg194Trp polymorphism in glioma risk based on 4 case-control studies [24, 27–29], and the results indicated that there was no remarkable association between them [8]. Then the findings from a meta-analysis by Sun et al. based on 7 studies were not totally similar to the above-mentioned results [7]. In 2013, Li et al. conducted a meta-analysis of 5 case-control studies, which revealed that XRCC1 Arg194Trp polymorphism might associated with risk of glioma [10]. The following meta-analysis performed by Jiang et al. of 6 case-control studies indicated Arg194Trp polymorphism might have no influence on the susceptibility of glioma [9]. The fifth meta-analysis from Zhang et al [11] including 8 case-controls showed that Arg194Trp polymorphism increased the glioma risk. The meta-analysis from He et al in 2014 based on 8 studies showed Arg194Trp probably increased risk for glioma due to studies deviating from HWE in controls [16]. Another six meta-analyses indicated that Arg194Trp polymorphisms might contribute to genetic susceptibility to glioma in the Chinese population [12, 14, 17–20]. Almost all published meta-analyses suggested that more large-scale, well-designed and population-based studies were required for further evaluation [7–19].

Obviously, their results are inconsistent (Supplementary Table 1). Nowadays, 16 case-control studies and have yielded inconsistent results (Figure 2). Meta-analysis is a useful tool to resolve the inconsistent results from single study, which has been broadly applied in epidemiological field [40–44]. Hence, we undertook this comprehensive meta-analysis to provide an updated approach on the overall relationship. The cumulative analysis was also used to evaluate the result influenced by sample sizes. In the end, our meta-analysis of 16 case-control studies indicated that XRCC1 Arg194Trp polymorphism probably was associated with increased risk of glioma, and the cumulative analysis suggested that the non-significant association would be change to significant if the sample sizes were enough. The subgroups analyses indicated that XRCC1 Arg194Trp polymorphism elevated disease risk in Asians, but not associated in Caucasians.

The results of our meta-analysis are opposed to the conclusions from previously six meta-analyses in overall population [7–10, 15, 16], which all indicated there was no association between XRCC1 Arg194Trp polymorphism and glioma risk. The major reason is that our meta-analysis included more studies than them; therefore, our meta-analysis is the most comprehensive one currently. In order to explore the influence of sample size on overall estimation, we conducted cumulative meta-analysis, which showed that the association became significant and the results were changed with the sample size cumulated (Figure 3). For the subgroup analysis, the results were varied; this might be also attributed to the sample sizes. Hence, relevant studies should be performed to further identify this relationship. Moreover, evidence indicates that ethnic-specific variation, different health care and socioeconomic class might exert an effect on the incidence of glioma [45]. When we performed subgroup analysis based on ethnicity, the results indicated no association in Caucasians but significant correlation in Asians, that might be attributable to the ethnic-specific background or insufficient sample size in Caucasians. Third, the results can be influenced by violations or deviations in HWE [46], which can explain the reason why the results were different of violation in HWE and deviation in HWE of our meta-analysis. Fourth, the source of controls also can impact the results. Generally, the population - based controls is more representative than hospital - based controls. In our meta-analysis, the result of hospital - based controls was different from that of population - based controls, and the former was similar with overall results whereas the latter was significant under two genetic models. This might indicate that the results of our meta-analysis were not influenced much by control source. Finally, the heterogeneity existed in the meta-analysis.

Our meta-analysis also is similar with the previous eight meta-analyses [7, 10, 11, 13, 17–20]. Therefore, our meta-analysis confirmed their results. We can observe that the association became significant from the study by Wang et al [32] in 2012 (Figure 3), which was just the sixth included study of meta-analysis by Jiang et al. [9]. The meta-analyses by Zhang et al. [11], He et al. [14, 16], Feng et al. [12], Xu et al. [13], Li et al. [17], Qi et al. [18] and Li et al. [19] also included this study and all of these meta-analyses showed a significant association except the one by He et al. [16]. This proved the evidence that the result could be influenced by sample size. Hence, the next advantage of meta-analysis is that we performed the cumulative meta-analysis.

Our meta-analysis also has its limitations. First, the primary studies only provided data regarding Caucasians and Asians; therefore, more studies involving other ethnicities such as African should be conducted to validate our results. Besides, the important information such as histological types should be improved in further studies. Second, other factors that might contribute to the heterogeneity, such as age, histological types, gender could not be explored due to the lack of relevant data were in original studies. Third, for lacking recommended quality assessment tool [44], it was difficult to assess the quality of included studies and its influence; these potential biases might result in lack of replication of definite conclusions. Fourth, although no publication bias was detected and we tried our best to identify relevant publications, due to the limitations of languages and using permission of databases, only studies published in English and Chinese were included. So several databases were not searched and the studies in other languages were omitted, which might result in the occurrence of selection bias. Finally, this meta-analysis merely detected the association between the XRCC1 Arg194Trp polymorphism and glioma based on crude data. Therefore, the effects of gene - gene and gene - environment interactions were not mentioned in this research.

In conclusion, the results of the present meta-analysis suggest that the XRCC1 Arg194Trp polymorphism is associated with increased risk of glioma, especially for Asians. Of course, the results of our meta-analysis should be treated with caution, but unlike previous meta-analyses, we do not need to emphasize that relevant studies should be carried out for enlarging the sample sizes.

## MATERIALS AND METHODS

This meta-analysis was reported following the recommended Preferred Reporting Items for Systematic Reviews and Meta-Analyses (PRISMA) statement [47].

### Eligibility criteria

The study was included according to the following criteria: (1) the study assessed the association between the glioma and XRCC1 Arg194Trp polymorphism; (2) the study reported ORs and 95%CIs, or the number of individual genotypes in both case and control groups for their calculation; (3) the design was case-control or cohort study; and (4) the patients were microscopically diagnosed as glioma. In addition, if studies had the overlapping data, only the largest or the most complete one was included in the final analysis.

### Search strategy

The PubMed, Embase, CBM (chinese biomedical database), and CNKI (China National Knowledge Internet) databases were comprehensively searched up to August 13, 2016 (updated on December 22, 2016) using the following terms: (polymorphism OR mutation OR variant) AND (glioma OR “brain tumor” OR glioblastoma OR “glial cell tumors” OR “brain neoplasms”) AND (XRCC1 OR “x-ray cross complementing group 1”). Additional studies were manually searched from the references of all identified studies and the recent reviews.

### Data extraction

Two authors selected studies according to the criteria listed above and extracted information from all eligible studies independently. The essential information contained the first author’s name, year of publication, country of origin, ethnicity of subjects, source of control, genotyping method, number of cases and controls and genotype frequency, ORs and its 95%CIs, and HWE for controls. All disagreements were resolved by consulting with a third author.

### Data analysis

First, the heterogeneity among included studies was detected using *I*^2^ statistics [48]. The value of *I*^2^ ≤ 40% was considered no substantive heterogeneity existed, so the fixed effect model was employed; otherwise, the random-effects model was used [49]. The OR and 95% CI were calculated for estimating the association between XRCC1 Arg194Trp polymorphism and glioma under the following common used five genetic models [50–53]: allele comparison (Trp vs. Arg), homozygote comparison (TrpTrp vs. ArgArg), heterozygote comparison (ArgTrp vs. ArgArg), dominant model (TrpTrp+ArgTrp vs. ArgArg), and recessive model (TrpTrp vs. ArgArg+ArgTrp), respectively. The subgroups analyses based on the ethnicity, source of controls, and the HWE for controls were performed to explore the potential source of heterogeneity among studies. Sensitivity analysis was applied by excluding each single study each time to explore the stability of overall results. The cumulative meta-analysis was carried out to observe the change when with sample sizes were enlarged [22, 23]. The publication bias was detected by funnel plot analysis and the Egger linear regression test [54]. All the analyses were performed using the Comprehensive Meta-Analysis software, version 2.2 (Biostat, Englewood, New Jersey) [50, 52, 53] and all the *p* values were two-sided.

## SUPPLEMENTARY MATERIALS FIGURES AND TABLES




