# Supplementary material for: Reappraisal of XRCC1 Arg194Trp polymorphism and glioma risk: a cumulative meta-analysis
Source: Oncotarget. 2017 Feb 16;8(13):21599–608. doi: 10.18632/oncotarget.15376 (PMC5400609; doi:10.18632/oncotarget.15376)
Supplement: Supplementary file 1 [file oncotarget-08-21599-s001.pdf]

Reappraisal of XRCC1 Arg194Trp polymorphism and glioma risk: a cumulative meta-analysis

SUPPLEMENTARY FIGURES AND TABLE

Study name	Cumulative statistics				
	Point	Lower limit	Upper limit	Z-Value	p-Value
Liu 2007	1.02	0.88	1.20	0.31	0.76
Kiuru 2008	1.00	0.88	1.15	0.06	0.95
Liu 2009	1.00	0.88	1.14	-0.04	0.97
Mckean-Cowdin 2009	1.00	0.90	1.11	-0.07	0.95
Rajaraman 2010	0.97	0.88	1.08	-0.55	0.58
Custodio 2011	1.11	0.82	1.51	0.70	0.48
Hu 2011	1.16	0.88	1.52	1.05	0.29
Zhou 2011	1.14	0.90	1.45	1.10	0.27
Wang 2012	1.13	0.93	1.37	1.19	0.23
Liu 2012	1.18	0.97	1.44	1.63	0.10
Luo 2013	1.19	0.99	1.42	1.86	0.06
Pan 2013	1.20	1.02	1.42	2.14	0.03
Xu 2014	1.19	1.03	1.38	2.35	0.02
Gao 2014	1.19	1.04	1.37	2.49	0.01
Li 2014	1.17	1.03	1.34	2.44	0.01
Fan 2016	1.18	1.05	1.34	2.67	0.01
	1.18	1.05	1.34	2.67	0.01

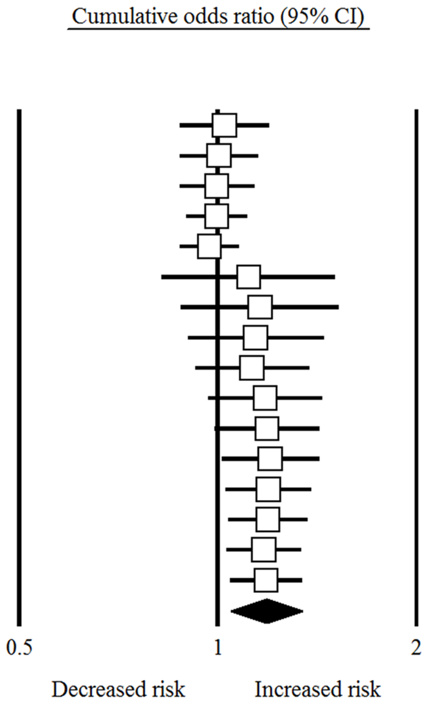

Supplementary Figure 1: Forest plot for cumulative analysis in Trp vs. Arg comparison.

Study name	Cumulative statistics				
	Point	Lower limit	Upper limit	Z-Value	p-Value
Liu 2007	1.02	0.82	1.26	0.19	0.85
Kiuru 2008	0.97	0.82	1.15	-0.35	0.73
Liu 2009	0.97	0.82	1.14	-0.38	0.70
Mckean-Cowdin 2009	0.98	0.86	1.11	-0.33	0.74
Rajaraman 2010	0.95	0.84	1.07	-0.85	0.40
Custodio 2011	1.16	0.80	1.70	0.79	0.43
Hu 2011	1.18	0.84	1.66	0.95	0.34
Zhou 2011	1.14	0.85	1.53	0.89	0.38
Wang 2012	1.10	0.86	1.41	0.77	0.44
Liu 2012	1.12	0.89	1.41	0.99	0.32
Luo 2013	1.10	0.89	1.35	0.89	0.37
Pan 2013	1.11	0.91	1.34	1.05	0.30
Xu 2014	1.41	1.01	1.97	2.01	0.04
Gao 2014	1.37	1.00	1.88	1.98	0.05
Li 2014	1.34	1.01	1.79	2.00	0.05
Fan 2016	1.34	1.02	1.77	2.09	0.04
	1.34	1.02	1.77	2.09	0.04

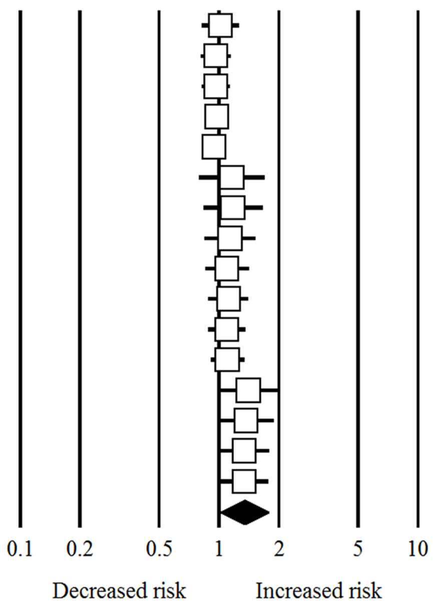

Supplementary Figure 2: Forest plot for cumulative analysis in ArgTrp vs. ArgArg comparison.

Study name	Cumulative statistics				
	Point	Lower limit	Upper limit	Z-Value	p-Value
Liu 2007	1.03	0.84	1.26	0.26	0.80
Kiuru 2008	0.99	0.84	1.16	-0.16	0.87
Liu 2009	0.98	0.84	1.15	-0.24	0.81
Mckean-Cowdin 2009	0.99	0.87	1.11	-0.23	0.82
Rajaraman 2010	0.96	0.85	1.08	-0.75	0.45
Custodio 2011	1.18	0.82	1.71	0.89	0.37
Hu 2011	1.22	0.87	1.70	1.15	0.25
Zhou 2011	1.18	0.89	1.58	1.15	0.25
Wang 2012	1.15	0.90	1.47	1.13	0.26
Liu 2012	1.19	0.95	1.50	1.48	0.14
Luo 2013	1.18	0.96	1.46	1.56	0.12
Pan 2013	1.19	0.98	1.45	1.77	0.08
Xu 2014	1.17	0.99	1.39	1.83	0.07
Gao 2014	1.17	0.99	1.37	1.89	0.06
Li 2014	1.16	0.99	1.34	1.89	0.06
Fan 2016	1.17	1.01	1.35	2.11	0.04
	1.17	1.01	1.35	2.11	0.04

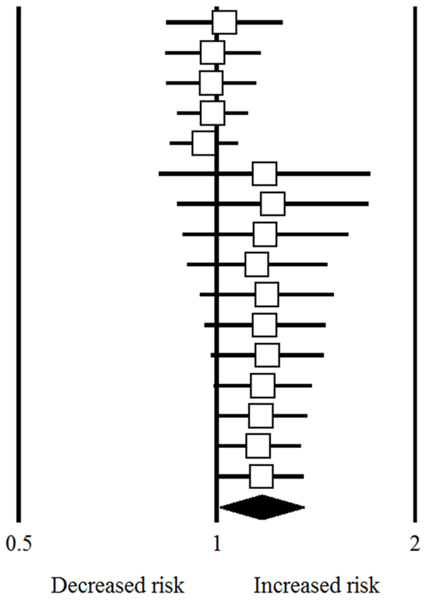

Supplementary Figure 3: Forest plot for cumulative analysis in TrpTrp+ArgTrp vs. ArgArg comparison.

Study name	Cumulative statistics				
	Point	Lower limit	Upper limit	Z-Value	p-Value
Liu 2007	1.04	0.74	1.46	0.24	0.81
Kiuru 2008	1.08	0.78	1.51	0.48	0.63
Liu 2009	1.07	0.77	1.48	0.41	0.68
Mckean-Cowdin 2009	1.07	0.78	1.47	0.39	0.70
Rajaraman 2010	1.06	0.77	1.45	0.34	0.73
Custodio 2011	1.18	0.89	1.57	1.16	0.25
Hu 2011	1.25	0.96	1.62	1.68	0.09
Zhou 2011	1.24	0.97	1.59	1.71	0.09
Wang 2012	1.27	1.01	1.59	2.04	0.04
Liu 2012	1.40	1.13	1.72	3.12	0.00
Luo 2013	1.46	1.20	1.77	3.75	0.00
Pan 2013	1.49	1.23	1.80	4.13	0.00
Xu 2014	1.53	1.29	1.82	4.83	0.00
Gao 2014	1.53	1.30	1.81	5.01	0.00
Li 2014	1.47	1.25	1.73	4.64	0.00
Fan 2016	1.47	1.26	1.72	4.86	0.00
	1.47	1.26	1.72	4.86	0.00

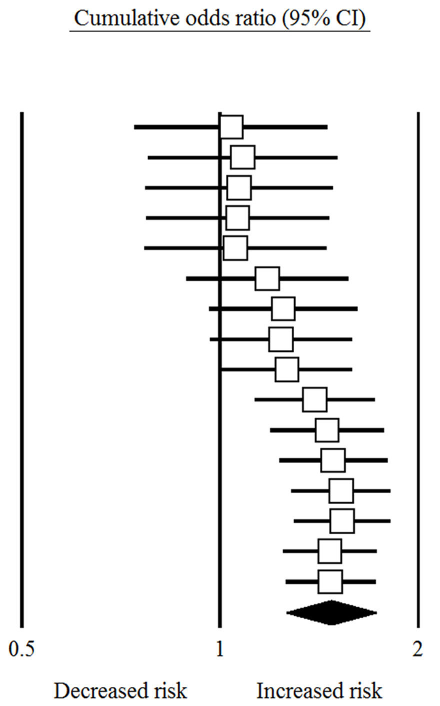

Supplementary Figure 4: Forest plot for cumulative analysis in TrpTrp vs. ArgArg+ArgTrp comparison.

Supplementary Table 1: Characteristics of published 14 meta-analyses.

Supplementary File 1
