# Supplementary material for: Reappraisal of XRCC1 Arg194Trp polymorphism and glioma risk: a cumulative meta-analysis
Source: Oncotarget. 2017 Feb 16;8(13):21599–608. doi: 10.18632/oncotarget.15376 (PMC5400609; doi:10.18632/oncotarget.15376)
Supplement: Supplementary file 2 [file oncotarget-08-21599-s002.doc]

**Supplemental table 1. Characteristics of published 14 meta-analyses**

| References | No. of studies | Included studies | Journal of publication | OR (95%CI) | Authors' conclusion |
| --- | --- | --- | --- | --- | --- |
| Zhang 2012 | 4 | Kiuru 2008; Rajaraman 2010; Hu 2011; Zhou 2011 | Exp Ther Med | Trp vs. Arg: 1.01(0.77-1.33)  TrpTrp vs. ArgArg: 1.56(0.96-2.54)  TrpTrp+TrpArg vs. ArgArg: 0.98(0.74-1.31)  TrpTrp vs. TrpArg+ArgArg: 1.48(0.92-2.38) | No association |
| Li 2013 | 5 | Kiuru 2008; Rajaraman 2010; Hu 2011; Zhou 2011; Custodio 2012 | J Neurooncol | TrpTrp vs. ArgArg: 2.208(1.099-4.435)  TrpTrp+TrpArg vs. ArgArg: 1.249(0.942-1.656) | No association |
| Jiang 2013 | 6 | Kiuru 2008; Liu 2009; Mckean-Cowdin 2009; Rajaraman 2010; Zhou 2011; Wang 2012 | PLoS ONE | Trp vs. Arg: 1.00(0.89–1.13)  TrpTrp vs. ArgArg: 1.36(0.89-2.09)  ArgTrp vs. ArgArg: 0.90(0.75–1.08)  TrpTrp+TrpArg vs. ArgArg: 0.98(0.86–1.11)  TrpTrp vs. TrpArg+ArgArg: 1.35(0.88–2.06) | No association |
| Sun 2012 | 7 | Kiuru 2008; Liu 2009; Mckean-Cowdin 2009; Rajaraman 2010; Hu 2011; Zhou 2011; Custodio 2012 | Asian Pac J Cancer Prev | Trp vs. Arg: 1.159(0.843-1.387)  TrpTrp vs. ArgArg: 2.029(1.398-2.945)  ArgTrp vs. ArgArg: 1.011(0.884-1.156)  TrpTrp+TrpArg vs. ArgArg: 1.232(0.840-1.809) | No association |
| Gu 2013 | 8 | Liu 2007; Kiuru 2008; Liu 2009; McKean-Cowdin 2009; Rajaraman 2010; Zhou 2011; Hu 2011; Custodio 2011 | Neural Regen Res | Trp vs. Arg: 1.16(0.88-1.52)  TrpTrp vs. ArgArg: 1.68(0.94-3.00)  TrpTrp+TrpArg vs. ArgArg: 1.21(0.87-1.70)  TrpTrp vs. TrpArg+ArgArg: 1.22(0.95-1.55) | No association |
| Zhang 2013 | 8 | Kiuru 2008; Liu 2009; Rajaraman 2010; Hu 2011; Zhou 2011; Custodio 2012; Wang 2012; Liu 2012 | Tumour Biol | TrpTrp vs. ArgArg: 2.03(1.32–3.12)  ArgTrp vs. ArgArg: 1.23 (0.88–1.72)  TrpTrp+TrpArg vs. ArgArg: 1.29(0.92–1.80)  TrpTrp vs. TrpArg+ArgArg: 1.72(1.31–2.25) | Significant association |
| He LW 2014 | 8 | Hu 2011; Zhou 2011; Wang 2012; Liu 2012; Luo 2013; Pan 2013; Xu 2013; Gao 2014 | PLoS ONE | Trp vs. Arg: 1.23(1.13–1.33)  TrpTrp vs. ArgArg: 1.82(1.48–2.25)  ArgTrp vs. ArgArg: 1.08(0.97–1.20)  TrpTrp+TrpArg vs. ArgArg: 1.17(1.06–1.30)  TrpTrp vs. TrpArg+ArgArg: 1.78(1.44–2.19) | Significant association in Asians |
| He H 2014 | 8 | Kiuru 2008; Liu 2009; McKean-Cowdin 2009; Rajaraman 2010; Custodio 2011; Hu 2011; Zhou 2011; Wang 2012 | Progress in Modern Biomedicine | TrpTrp vs. ArgArg: 1.83(1.32-2.52) | Significant association in deviated from HWE studies |
| Feng 2014 | 11 | Kiuru 2008; Liu 2009; McKean-Cowdin 2009; Rajaraman 2011; Custodio 2011; Hu 2011; Zhou 2011; Wang 2012; Liu 2012; Luo 2013; Pan 2013 | Tumour Biol | TrpTrp vs. ArgArg: 2.03 (1.61–2.57)  TrpTrp vs. TrpArg+ArgArg: 1.77 (1.41–2.22) | Significant association in Asians |
| Xu 2014 | 12 | Kiuru 2008; Liu 2009; McKean-Cowdin 2009; Rajaraman 2011; Custodio 2011; Hu 2011; Zhou 2011; Wang 2012; Liu 2012; Luo 2013; Pan 2013; Xu 2013 | Asian Pac J Cancer Prev | Trp vs. Arg: 1.259(1.045-1.517)  TrpTrp vs. ArgArg: 2.108(1.593-2.789)  ArgTrp vs. ArgArg: 1.106(0.901-1.359)  TrpTrp+TrpArg vs. ArgArg: 1.23(0.997-1.519)  TrpTrp vs. TrpArg+ArgArg: 1.918(1.575-2.336) | Significant association |
| Li 2015 | 14 | Wang 2012; Rajaraman 2010; Kiuru 2008; Xu 2013; Liu 2009; Hu 2011; Zhou 2011; McKean-Cowdin 2009; Custódio 2011; Liu 2012; Pan 2013; Luo 2013; Li 2014; Gao 2014 | Int J Clin Exp Med | Arg vs. Trp: 0.72 (0.55-0.93)  ArgArg vs. TrpTrp: 0.55 (0.46-0.67)  ArgTrp vs. TrpTrp: 0.81 (0.58-1.13)  ArgArg vs. TrpTrp+TrpArg: 0.64 (0.45-0.91)  TrpArg+ArgArg vs. TrpTrp: 0.61 (0.51-0.74) | Significant association in Asians |
| Qi 2016 | 11 | McKean-Cowdin 2009; Rajaraman 2010; Custódio 2011; Hu 2011; Zhou 2011; Wang 2012; Liu 2012; Pan 2013; Gao 2014; Li 2014; Xu 2014 | Mol Neurobiol | Trp vs. Arg: 1.22 (0.99-1.46)  TrpTrp vs. ArgArg: 1.74 (1.29-2.34)  ArgTrp vs. ArgArg: 1.07 (0.98-1.17)  TrpTrp+TrpArg vs. ArgArg: 1.23 (1.00-1.51)  TrpTrp vs. TrpArg+ArgArg: 1.59 (1.30-1.94) | Significantly raises the risk of glioma for Asian |
| Li 2016 | 9 | Wang 2012; Xu 2013; Hu 2011; Zhou 2011; Liu 2012; Pan 2013; Luo 2013; Li 2014; Gao 2014 | Chinese Journal of Experimental Surgery | Arg vs. Trp: 0.83(0.77-0.90)  ArgArg vs. TrpTrp: 0.59(0.48-0.72)  ArgArg vs. TrpTrp+TrpArg: 0.86(0.79-0.95)  TrpArg+ArgArg vs. TrpTrp: 0.61 (0.50-0.74) | Significantly increased the risk of glioma in Asian |
| Liu 2016 | 13 | Rajaraman 2010; Kiuru 2008; Xu 2013; Liu 2009; Hu 2011; Zhou 2011; McKean-Cowdin 2009; Custódio 2011; Liu 2012; Pan 2013; Luo 2013; Li 2014; Gao 2014 | Cell Mol Neurobiol | Arg vs. Trp: 0.822 (0.699–0.966)  ArgArg vs. TrpTrp+TrpArg: 0.830 (0.688–1.001)  TrpArg+ArgArg vs. TrpTrp: 0.606 (0.500–0.734) | Might bring about higher risk of glioma in Asian population |
